# CRNDE: an oncogenic long non-coding RNA in cancers

**DOI:** 10.1186/s12935-020-01246-3

**Published:** 2020-05-12

**Authors:** Ya Lu, Huanhuan Sha, Xun Sun, Yuan Zhang, Yang Wu, Junying Zhang, Hui Zhang, Jianzhong Wu, Jifeng Feng

**Affiliations:** 1 Nanjing Medical University Affiliated Cancer Hospital & Jiangsu Cancer Hospital & Jiangsu Institute of Cancer Research, Baiziting42, 210009 Nanjing, China; 2 The Forth Clinical School of Nanjing Medical University, Nanjing, China

**Keywords:** CRNDE, lncRNA, Cancer, Biomarker

## Abstract

Colorectal neoplasia differentially expressed (CRNDE) is a long non-coding RNA which has been proved upregulated in various cancers. Meanwhile, CRNDE has been demonstrated to be involved in multiple biological processes of different cancers according to previous study. Moreover, recent studies suggested CRNDE might be a potential diagnostic biomarker and prognostic predictor due to its high sensitivity and specificity in cancer tissues and plasma. In this review, we summarize the biological function of CRNDE and the relevant mechanisms in cancers to establish a molecular basis for the clinical use of CRNDE in the future.

## Background

Long non-coding RNAs (lncRNAs) are a class of non-protein-coding RNAs that are more than 200 nucleotides in length and are involved in numerous physiological and pathological processes [1]. Multiple lines of evidence have shown dysregulated lncRNAs are associated with human cancers [2, 3]. LncRNAs regulate genes expression at the epigenetic, transcriptional and post-transcriptional levels [4, 5], and participate in a variety of molecular regulatory mechanisms. Such as inducing chromatin remodelling [6] and histone modification [7, 8], modulating alternative splicing patterns, acting as small RNA precursors [9], generating endo-siRNAs [10, 11], interfering transcription [12], mediating protein activity [13], altering protein localization [14], and exerting structural or organizational activities [15]. Thus, it is becoming increasingly clear that long non-coding RNAs play indispensable roles in human cancers.

CRNDE is located at an atypical locus—hCG_1815491 on chromosome 16—and is activated early in colorectal cancer (CRC) [16]. CRNDE is classified as a long non-coding RNA because it is longer than 200 nucleotides and is independent of any known protein-coding genes. In addition, at least 10 splice variants of CRNDE are expressed, among which CRNDE-a, -b, -d, -e, -f, -h, and -j are the most abundant isoforms [16, 17]. Although CRNDE was originally identified to be specifically high-expressed in CRC [17], overexpressed CRNDE was also observed in other cancers, such as glioma [18, 19], hepatocellular carcinoma (HCC) [20–24], lung cancer [25, 26], breast cancer (BC) [27], gastric cancer (GC) [28, 29], and renal cell carcinoma (RCC) [30, 31]. CRNDE was considered to play crucial roles in cancer biological processes and act as a key factor to affect multiple molecular regulatory networks.

Here, we will summarize the specific biological processes that CRNDE influencing in and the diverse molecular mechanisms that CRNDE involving in, to provide the theoretical basis for CRNDE practical usage in clinic (Table 1).

**Table 1:** The related interactive genes of CRNDE and involvement in functional role in different cancers

Functional roles	Cancer types	Related genes	Related pathways	References
Proliferation	CRC	miR-217/TCF7L2, hnRNPUL2, miR-181a-5p/beta-catenin/TCF4, EZH2/DUSP5/CDKN1A	Wnt/beta-catenin, Ras/MAPK signaling pathway	[32, 33, 35, 48]
	Glioma	miR-384/PIWIL4, miR-136-5p/Bcl-2/Wnt2, miR-186/XIAP/PAK7	mTOR signaling pathway	[19, 39–41]
	Lung caner	miR-338-3p, CDK4, CDK6, CCNE1	PI3K/AKT signaling pathway	[25, 26]
	HCC	miR-136-5P/IRX5,miR-217/MAPK1, miR-203/BCAT1, miR-384/NF-kappaB/p-AKT, E-cadherin, ZO-1, N-cadherin, slug, twist, vimentin	PI3K/Akt/GSK3beta-Wnt/beta-catenin, mTOR signaling pathway	[20–24, 38, 77]
	BC	miR-136, SF3B1	Wnt/beta-catenin signaling pathway	[27, 78]
	GC	miR-145/E2F3	PI3K/AKT signaling pathway	[28, 29]
	RCC	CCND1, CCNE1	Wnt/beta-catenin signaling pathway	[31, 79]
	Osteosarcoma	Notch1, JAG1, N-cadherin, vimentin, E-cadherin	Notch1 signaling pathway	[36]
	Cervical cancer	Not determined	PI3K/AKT signaling pathway	[34, 80]
	Ovarian cancer	TP53	Not determined	[66]
	MM	miR-451	Not determined	[47]
	PTC	miR-384/PTN	Not determined	[43]
	Pancreatic cancer	miR-384/IRS1	Not determined	[50]
	TSCC	miR-384/KRAS/cdc42	Not determined	[42]
	Melanoma	miR-205/CCL18, SF3B1	Not determined	[81, 82]
	Medulloblastoma	Not determined	Not determined	[83]
	Bladder cancer	Not determined	Not determined	[68]
Invasion and migration	CRC	miR-136/E2F1, miR-217/TCF7L2, hnRNPUL2	Wnt/beta-catenin, Ras/MAPK Signaling Pathway	[32, 35, 49]
	Glioma	miR-384/PIWIL4/STAT3, miR-136-5p/Bcl-2/Wnt2, miR-186/XIAP/PAK7	mTOR signaling pathway	[19, 39–41]
	Lung caner	miR-338-3p	Not determined	[25]
	HCC	miR-136-5P/IRX5, miR-217/MAPK1, miR-203/BCAT1, miR-384/NF-kappaB/p-AKT,E-cadherin, ZO-1, N-cadherin, slug, twist, vimentin	Wnt/beta-catenin signaling pathway	[20–22, 24, 38]
	BC	miR-136	Wnt/beta-catenin signaling pathway	[27]
	GC	Not determined	PI3K/AKT signaling pathway	[28]
	Osteosarcoma	Notch1, JAG1, N-cadherin, vimentin, E-cadherin	Notch1 signaling pathway	[36]
	Cervical cancer	Not determined	PI3K/AKT signaling pathway	[80]
	PTC	miR-384/PTN	Not determined	[43]
	Pancreatic cancer	miR-384/IRS1	Not determined	[50]
	TSCC	miR-384/KRAS/cdc42	Not determined	[42]
	Melanoma	miR-205/CCL18	Not determined	[81]
	Gallbladder cancer	DMBT1/C-IAP1	PI3K-AKT signaling pathway	[76]
	Bladder cancer	Not determined	Not determined	[68]
Apoptosis	CRC	EZH2/DUSP5/CDKN1A	Not determined	[48]
	Glioma	miR-384/PIWIL4/STAT3, EGFR/TKI, Bcl2/Bax, miR-136-5p/Bcl-2/Wnt2, miR-186/XIAP/PAK7, FOXM1	EGFR, NF-kappaB, JAK/STAT signaling pathway	[39–41, 46, 58]
	HCC	Not determined	mTOR signaling pathway	[77]
	Cervical cancer	Not determined	PI3K/AKT signaling pathway	[34]
	MM	miR-451	Not determined	[47]
	Medulloblastoma	Not determined	Not determined	[83]
	Bladder cancer	Not determined	Not determined	[68]
Chemoresistance	CRC	miR-181a-5p/beta-catenin/TCF4, miR-136/E2F1	Wnt/beta-catenin signaling pathway	[33, 49]
Metabolism	CRC	IGF	PI3K/Akt/mTOR, Raf/MAPK, insulin/IGF signaling pathway	[74]
Radiosensitivity	Lung caner	PRC2/EZH2/p21	Not determined	[64]
Inflammation	Glioma	FOXM1	NF-kappaB, JAK/STAT, toll-like receptor (TLR) signaling pathway	[58, 59]

## CRNDE promotes proliferation

Numerious studies revealed overexpressed CRNDE significantly promoted cancer cells proliferation. Specifically, diverse signaling pathways were found associated with CRNDE effect in cancers, such as the Wnt/β-catenin [20, 31–33], PI3K/AKT/mTOR [23, 26, 34], Ras/mitogen-activated protein kinase (MAPK) [35] and Notch1 signaling pathways [36]. Among them, the Wnt/β-catenin signaling pathway could be directly activated when CRNDE promoted BC cells proliferation by repressing the expression of miR-136. Meanwhile, in this study, miR-136 was considered as a binding target of CRNDE and along with the levels of β-catenin, c-myc and cyclin D1 were increased by upregulaed CRNDE [27]. While, Tang et al. [23] demonstrated CRNDE could exert its oncogenic role in HCC cells growth via mediating the PI3K/AKTGSKβ-Wnt/β-catenin axis. Moreover, CRNDE was reported to competitive bind with miR-217 [32] and miR-181a-5p [33], increasing Wnt/β-catenin signaling activity to participate in different cancer cells proliferation. At the same time, the expression levels of downstream target genes of these two microRNAs, TCF7L2 [32] and TCF-4 [33], were increased. Collectively, the results above indicated that Wnt/β-catenin signaling might be the key pathway through which CRNDE could exert its cancer-promoting function in various cancers [37]. Furthermore, CRNDE was found be able to form a functional complex with heterogeneous nuclear ribonucleoprotein U-like 2 protein (hnRNPUL2) and direct the transport of hnRNPUL2 between the nucleus and cytoplasm [35]. Cytoplasmic aggregation hnRNPUL2 simultaneously enhanced the stability of CRNDE at the RNA level and on the other hand, CRNDE depletion downregulated a series of downstream genes involved in the Ras/MAPK signaling pathway in CRC [35].

Clearly, lncRNAs are inextricably linked with microRNAs, and lncRNA CRNDE is no exception. An increasing number of studies have shown that CRNDE could act as a competitive endogenous RNA (ceRNA) or molecular sponge to target some certain microRNAs, thus inducing the proliferation of different cancers. For example, CRNDE could accelerated non-small-cell lung cancer (NSCLC) progression by sponging miR-338-3p [25] and enhanced HCC carcinogenesis by sponging miR-203, miR-384 or miR-217, thereby mediating BCAT1, NF-κB, p-AKT and MAPK1 expression [22, 24, 38]. Moreover, in glioma, miR-136-5p, miR-384 and miR-186 could be negatively regulated by CRNDE to facilitate cancer cells growth [39–41]. Special findings were reported that CRNDE overexpression in glioma resulted in decreased protein level of piwi-like RNA-mediated gene silencing 4 (PIWIL4). Not only was PIWIL4 regulated by miR-384, but the downstream proteins of PIWIL4—STAT3, cyclin D1, VEGFA, SLUG and MMP-9 were also modulated by miR-384 [40]. Similarly, CRNDE also could stimulate the development of tongue squamous cell carcinoma (TSCC) by inhibiting miR-384 expression [42], accelerate the progression of GC via molecular sponging of miR-145 [29] and activate carcinogenesis of papillary thyroid cancer (PTC) by suppressing miR-384 [43]. In addition to the competitive binding and sponge-like interactions between CRNDE and microRNAs, there might be other intricate molecular mechanisms by which CRNDE exerts its unique cancer-promoting effect. For instance, CRNDE impeded miR-136-5p expression in a RISC-dependent manner, and a reciprocal repression feedback loop following formed between CRNDE and miR-136-5p [21]. On the other hand, IRX5, which has been confirmed to be the neighbouring mRNA of CRNDE, enhanced the tumorigenicity of HCC cells as a potential downstream target gene of miR-136-5p. MiR-136-5p coud interact with 3′ UTR of IRX5 and regulate its expression. Thus, according to this study, CRNDE exhibited oncogenic properties via CRNDE–miR-136-5P–IRX5 axis in human HCC [21].

**Fig. 1:**
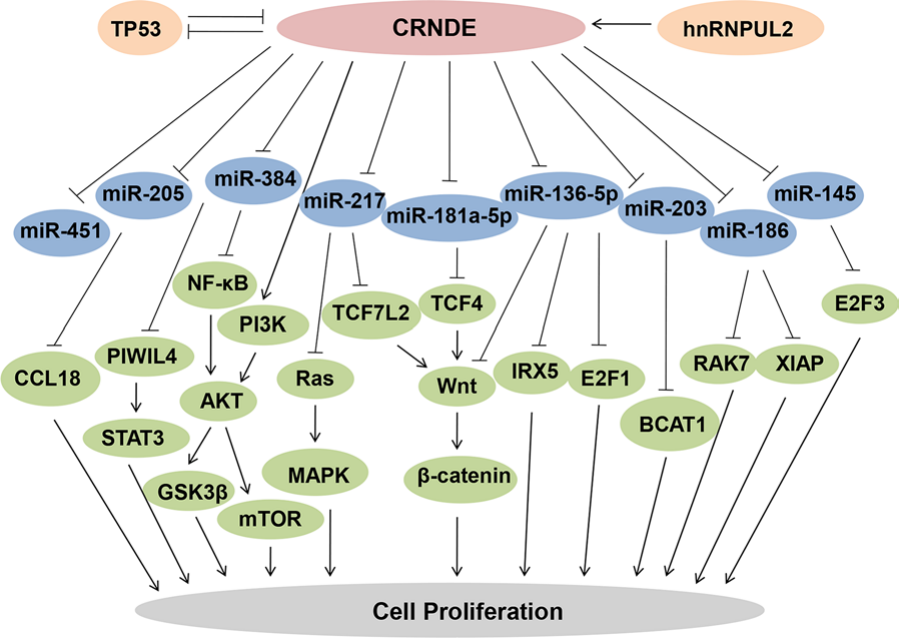
CRNDE significantly promotes cell proliferation through mediating multiple signaling pathways and various target genes. Among them, the most important pathways are Wnt/β-catenin, PI3K/AKT, NF-κB/AKT and Ras/MAPK signaling pathway, and the most relevant target microRNAs including miR-181a-5p, miR-136-5p, miR-217, miR-384, miR-203, miR-186, miR-205, miR-145 and miR-451. Moreover, the accumulation of TP53 is correlated with decreased expression of CRNDE, while the accumulation of hnRNPUL2 in the cytoplasm increases the stability of CRNDE

Consequently, CRNDE, as a crucial tumor promoter, facilitates the progression of different cancers through a variety of molecular pathways. Overexpression of CRNDE promotes cell growth and proliferation, increases the proportion of cells in proliferative subpopulations (S phase) while decreasing the proportion of cells in quiescent subpopulations (G0/G1 phase), and modulates the expression of CDK4, CDK6, CCDN1 and CCNE1 [26, 31]. Collectively, the above findings show the importance of CRNDE in the process of cancer cells proliferation (Fig. 1).

## CRNDE inhibits apoptosis

Apoptosis is a mode of programmed cell death that results in the precise, orderly and efficient removal of damaged cells [44]. Deregulation of apoptotic cell death process is a hallmark of cancer, alterations in apoptosis are responsible for cancer genesis and development and thus may play a vital role in the prognoses of cancers [45]. In the light of previous reports, CRNDE was considered assocoated with cell apoptosis process and thereby promoting cancer progression.

In glioma, knockdown CRNDE induced cancer cells apoptosis and then decreased Bcl2/Bcl2-associated X protein (Bax) ratio through different pathways [39, 40, 46]. Epidermal growth factor (EGF) enhanced the upregulation of CRNDE expression whereas epidermal growth factor receptor (EGFR) tyrosine kinase inhibitors (TKIs) blocked it, suggesting that EGFR activation promoted high expression of CRNDE, which in turn decreased the Bcl2/Bax ratio and subsequently inhibited apoptosis [46]. Moreover, CRNDE also acted as a ceRNA that bound to and negatively regulated miR-136-5p in glioma, subsequently protecting Bcl-2 and Wnt2 from miR-136-5p-mediated inhibition [39]. In addition, Bcl-2, Bcl-xL and caspase 3 were also the downstream of PIWIL4 and thence CRNDE could interfere with glioma cells apoptosis through CRNDE-miR-384-PIWIL4 axis [40]. Coincidentally, CRNDE also inhibited the apoptosis of glioma cells by decreasing XIAP and PAK7 expression by binding to and negatively regulating miR-186. Specifically, miR-186 bound to the 3′ UTR region of XIAP and PAK7, and further modulating their downstream proteins caspase 3, BAD, cyclin D1 and MARK2 [41]. Through the same mode of action, the anti-apoptotic activity of miR-451 was induced by CRNDE in multiple myeloma (MM) [47]. In fact, other complicated molecular mechanisms were involved, in addition to function by negatively targeting microRNAs. Yang et al. [34] proposed that depleted CRNDE prompted cervical cancer cells apoptosis through inactivating the PI3K/AKT pathway. While in CRC, CRNDE was shown to cause apoptosis by binding to EZH2 (the key component of polycomb repressive complex 2 (PRC2)) and epigenetically suppressing the expression of dual-specificity phosphatase 5 (DUSP5) and CDKN1A [48].

**Fig. 2:**
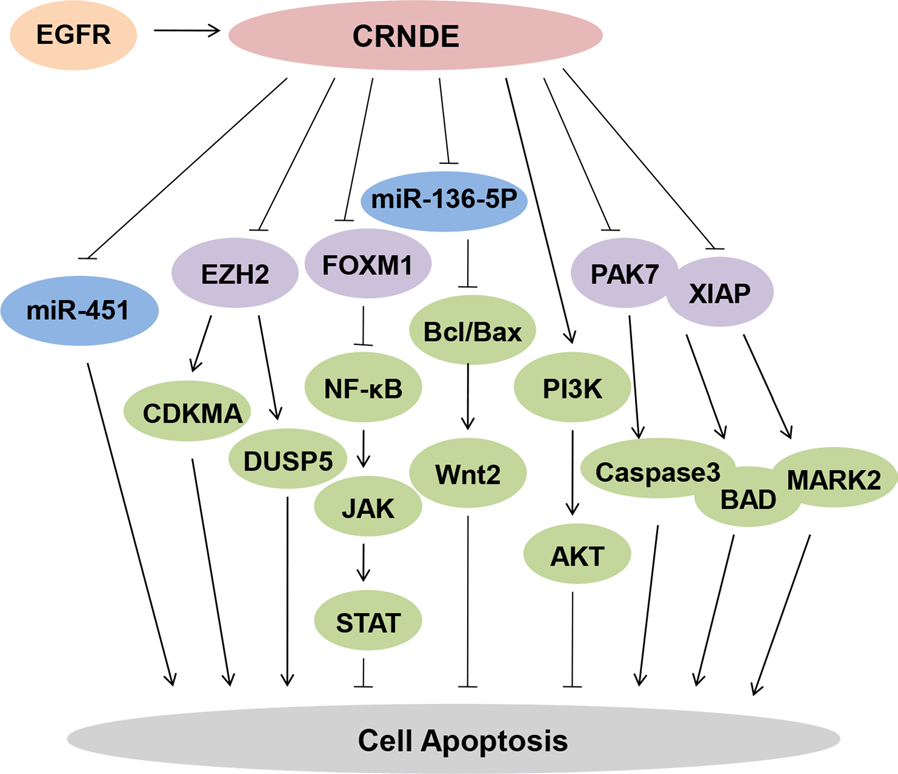
CRNDE prominently inhibits cell apoptosis by regulating several relevant effectors. In addition to the miR-384/PIWIL4/STAT3 axis, CRNDE directly regulates miR-136-5p, miR-451, EZH2, FOXM1, PAK7, XIAP and other genes to inhibit apoptosis as well. CRNDE is also involved in the controlling processes via caspase family, Bcl-2 family and some tumor suppressors, such as caspase3, Bcl-2, Bcl-xL, Bax, BAD, MARK2, CDKMA and DUSP5 et al., suppressing apoptosis

In summary, these results indicated that the dysregulation of CRNDE affectd various cancers apoptosis by targeting multiple genes, especially microRNAs. In a word, CRNDE influences the balance of interactions in apoptosis, and this dynamic equilibrium determines the final phenotype of CRNDE function in cancers (Fig. 2).

## CRNDE induces invasion and migration

Migration and invasion are the initial steps underlying cancer cell metastasis, which is a major contributor to the death of cancer patients. On the basis of our summary, the same target genes and signaling pathways were often involved in the biological process of CRNDE affecting both cancer cells proliferation, invasion and migration. As described above, CRNDE could inhibit miR-136 [21, 27, 49] and miR-217 [32] via sponging or competitive binding, activate Wnt/β-catenin signaling and heighten cell viability and metastatic activity [20]. Moreover, other microRNAs, such as miR-384 [38, 40, 42, 43, 50], miR-203 [24], miR-186 [41], and miR-338-3p [25], were all the negative targets of CRNDE and play important roles in promoting invasion and migration of a diverse range of cancers. For example, existing research suggested that CRNDE advanced the course of CRC cells metastasis by raising TCF7L2 expression and Wnt/β-catenin signaling activity through competitive binding to miR-217 [32]. In line with the study of Gao et al. [49], the competitive effect between CRNDE and miR-136 led to the disinhibition of the endogenous target gene E2F transcription factor 1 (E2F1) of miR-136, and then boosted the capability of metastasis in CRC.

Epithelial-mesenchymal transition (EMT) has been recognized as a critical biological process in cancer metastasis. The expanding knowledge relating to EMT has clarified the EMT programme as a set of dynamic transitional processes that shifts cells from epithelial to mesenchymal phenotypes. During this procedure, epithelial cells alter their morphology, motor their behaviour and acquire the ability for increased metastasis [51–53]. Therefore, EMT is an important contributor to cancer cells invasion and migration. In HCC, downregulated CRNDE suppressed the EMT process by increasing E-cadherin and ZO-1 expression while decreasing N-cadherin, slug, twist and vimentin expression and simultaneously restrained Wnt/β-catenin signaling [20]. Furthermore, in osteosarcoma, overexpression of CRNDE enhanced Notch1 signal transduction activity and promoted EMT programme, Notch1, JAG1, N-cadherin and vimentin were upregulated, while CRNDE knockdown produced the opposite effects [36]. Collectively, these results showed that CRNDE had the capacity to stimulate EMT so that cancer metastasis was advanced.

**Fig. 3:**
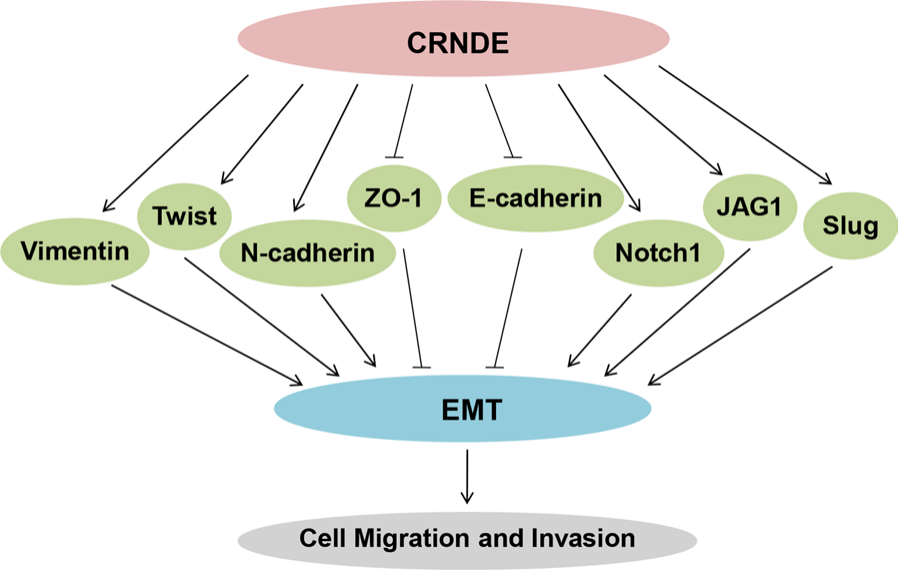
As for cell invasion and migration, CRNDE affects this process by multiple factors. CRNDE increases the ability of cell invasion and migration by modulating EMT process, and Notch1, JAG1, slug, vimentin, twist, N-cadherin, E-cadherin and ZO-1 are all involved in the EMT process. Those microRNAs that also play roles in cell proliferation are not repeated here

Last but not least, in addition to mediating Wnt/β-catenin and Notch1 signaling pathways, additional signaling pathways could be aberrantly activated or curbed, such as the NF-κB/AKT [38], Ras/MAPK [22, 35] and PI3K/AKT signaling pathways [28]. Sum up, CRNDE facilitates cancer cells invasion and migration by intervening multiple metastasis-related genes, highlighting the important role of CRNDE-mediated regulatory networks in cancer invasion and migration (Fig. 3).

## CRNDE regulates inflammatory responses

One of the most challenging issues in immunology remains to explore the latent mechanisms of the immune system in affecting the occurence and development of cancers. After years of research, it is appreciated that the immune system plays a dual role in cancer. Not only does it inhibit cancer growth by impairing cancer cells or reprogramming their end fate, but it also promotes cancer progression by selecting immunocompetent hosts that are more suitable for cancer cells survival [54, 55].

The NF-κB transcription factor family is believed to be the central mediator in the inflammatory process and the key participant in several immune responses [56]. DiDonato et al. summarized the complex role of NF-κB in linking inflammation with cancers and pointed out the harmful effects of insufficient or excessive NF-κB on cancers [57]. Thus, NF-κB has been confirmed to be a significant player in the immune process in which inflammation gradually evolves into cancers. CRNDE was reported to be upregulated in lipopolysaccharide (LPS)-injured WI-38 cells. The overexpressed CRNDE accelerated the cellular inflammation induced by LPS exposure and further activated the NF-κB and JAK/STAT signaling pathways [58]. Interestingly, in this molecular network, FOXM1 was upregulated by CRNDE, and in turn, FOXM1 depleration blocked the promoting effect of CRNDE in inflammatory responses [58]. Moreover, in another study [59], NF-κB and numerous cytokines were regulaed by CRNDE and the two were recognized as the effectors of the Toll-like receptor signaling pathway. Therefore, CRNDE was speculated as the inflammation trigger in astrocytes to affect tumorigenesis via the Toll-like receptor pathway, especially the Toll-like receptor-3 (TLR3)-mediated MyD88-independent pathway [59].

**Fig. 4:**
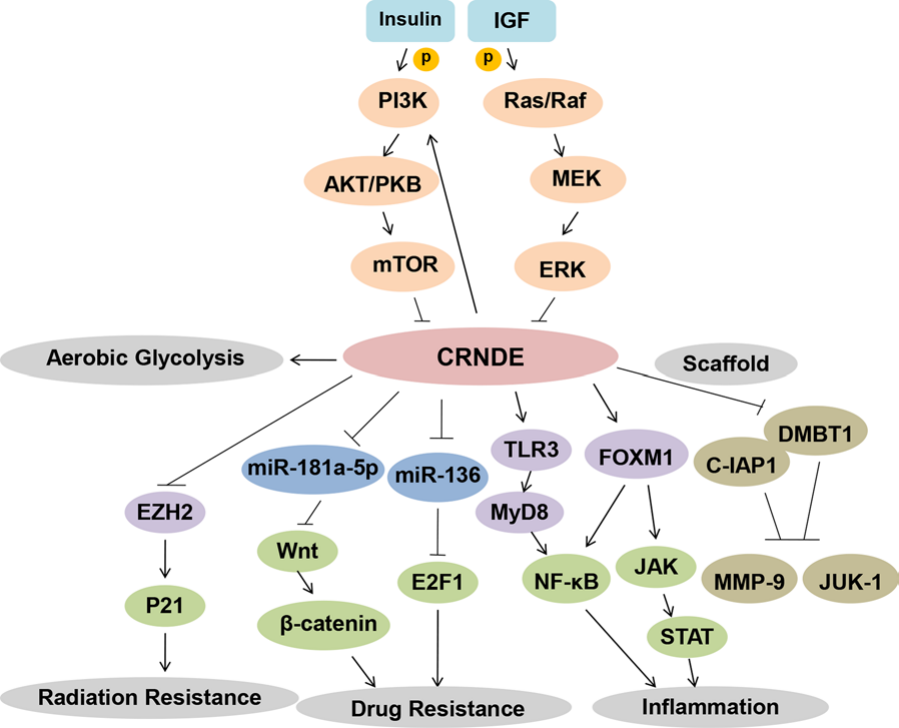
CRNDE influences drug resistance, radiation resistance and immune response, participants in glucose metabolism and also acts as a gene scaffold. CRNDE triggers inflammation to regulate tumorigenesis by targeting FOXM1 and TLR3/MyD8 axis, and activating NF-κB and JAK/STAT signaling pathways. Moreover, CRNDE leads to the formation of drug-resistance and radiation-tolerant phenotypes through interacting with EZH2, miR-181a-5p and miR-136. Furthermore, CRNDE can be regulated by insulin and IGFs through the PI3K/Akt/mTOR and the Raf/MAPK pathways to take part in aerobic glycolysis (Warburg effect). Especially, CRNDE functions as the scaffold of DMBT1 and C-IAP1 to help them exert their effect in cancers. All these illustrates that CRNDE play different important roles in the complicated networks of cancers

Currently, an increasing number of studies have deemed lncRNAs as important regulators of cancer immune system [60–62]. The in-depth studies of lncRNAs in tumor immunity have revealed the complicated molecular mechanisms in cancer immune system from a new perspective [63]. Thus, lncRNA CRNDE may be a potential target in cancer immunotherapy (Fig. 4).

## CRNDE affects chemoresistance and radioresistance

Chemotherapy and radiotherapy are conventional methods of cancer treatment with the basic principle of abolishing growing cancer cells and thus reducing the tumor burden. However, drug resistance and radiation resistance in cancer cells remain fundamental challenges. For both non-targeted and targeted drugs, intrinsic or acquired resistance in cancer cells is always a major obstacle in chemotherapy. As an oncogene, CRNDE has been acknowledged to induce cancer cells proliferation, trigger EMT, promote metastasis and regulate the immune microenvironment. On this basis, CRNDE was speculated whether could participate in weakening cancer chemosensitivity or radiosensitivity through unknown molecular mechanisms, ultimately conferring resistance. For instance, CRNDE was reported to influence CRC chemoresistance by modulating the expression of miR-181a-5p and the activity of the Wnt/β-catenin signaling pathway [33]. Specifically, in this research, CRC cell lines with stable CRNDE overexpression or miR-181a-5p knockdown were structured and were treated with serially increasing concentrations of 5-fluorouracil (5-Fu) and oxaliplatin (OXA). The upregulated CRNDE reduced CRC cells sensitivity to 5-Fu and OXA in varying degrees. Similarly, Gao et al. [49] found that downregulated CRNDE in combination with OXA treatment decreased cancer cells viability and promoted DNA damage and apoptosis, while CRC cells with upregulated CRNDE showed reduced DNA damage and apoptosis upon OXA treatment. The reason for which was CRNDE functioned as a ceRNA of miR-136 to promote drug resistance in CRC.

Radioresistance is also a difficult obstacle should be overcome in the treatment of cancers. Therefore, many in-depth studies aiming to explore the molecular mechanisms associated with radioresistance are being carried out. In a recent study, CRNDE was reported to influence the radiosensitivity of lung adenocarcinoma (LAD) by affecting the G1/S transition and leading to apoptosis [64]. Furthermore, CRNDE recruited the core component of PRC2, EZH2, to the p21 (CDKN1A) promoter region, ultimately contributing to the formation of radiation-tolerant phenotypes in LAD cells [64].

Berifly, CRNDE plays an important role in the molecular mechanisms of drug resistance and radiation tolerance and is expected to provide a new direction for further clinical treatment (Fig. 4).

## CRNDE serves as a potential tumor marker

CRNDE is currently confirmed to have 14 splice variants due to its complex splicing patterns, which result from various combinations of exons and introns [65]. Diverse splicing patterns generate different RNA transcripts, which may have different biological functions. CRNDE has been identified to be closely related to the malignant progression and poor prognosis of multiple cancers in different studies [18, 30, 66–69].

Dysregulation of universal splicing is a common feature of cancer cells, and some specific alternative splice variants of CRNDE have been shown to be related to cancers, especially CRC [67, 70, 71]. In a number of clinical sample-based studies, CRNDE expression was found to be higher in clinical cancer samples than that in normal tissue samples, and its upregulation was significantly correlated with larger tumor size, a greater degree of positive regional lymph node metastasis, faster distant metastasis and worse overall survival [16, 33, 35, 67, 70, 72]. Furthermore, on the basis of previous research, increased levels of CRNDE, especially CRNDE-h, -g and -b, in cancer tissue and blood were highly sensitive and specific for discriminating patients with CRC [16]. Similarly, Graham LD et al. [16] also demonstrated that CRNDE-h could effectively discriminate adenoma and normal mucosal tissue with a sensitivity and specificity of 95% and 96%, in a large number of clinical samples of CRC. In addition, CRNDE-b and -g showed 91% and 80% sensitivity, respectively, and the same 96% specificity as CRNDE-h within the same cohort. Whereas, in plasma, CRNDE-h RNA levels were characterized by 87% sensitivity and 93% specificity for discriminating CRC patients and healthy individuals.

Through numerous multi-angle quantitative analyses of clinical samples, not only in CRC but also in other solid tumors such as glioma [18, 19, 73], GC [28], NSCLC [25], BC [27], RCC [30], ovarian cancer [66], cervical cancer [34], and bladder cancer [68], CRNDE has been revealed to be closely associated with advanced stage and poor prognosis, suggesting that its promising significance in clinical application.

Thus, these data offer convincing evidences for CRNDE as a useful diagnostic marker and prognostic predictor in the future.

## Conclusions

This review aims to summarize the pleiotropic effects of CRNDE on the progression of various cancers. Many factors are involved in the regulation of CRNDE networks. In CRC, HCC, BC, GC, RCC, MM, glioma and other cancers, the expression levels of CRNDE are all higher in cancer tissues than that in corresponding normal tissues, indicating that CRNDE maybe serves as a cancer promoter. In addition, CRNDE participates in diverse cancer biological processes, including proliferation, apoptosis, invasion and metastasis, by regulating multiple target genes and affecting complicated signaling pathways. Meanwhile, the abnormal increase in CRNDE expression indicates the adverse prognosis and worse survival. Moreover, overexpressed CRNDE is also able to render cancer cells resistant to certain chemotherapeutic drugs and radiation, hindering the effective clinical treatment of cancers. In addition to playing a role in the biological processes described as above, CRNDE has been reported to be regulated by insulin and insulin-like growth factors (IGFs) through the PI3K/Akt/mTOR and Raf/MAPK pathways, thereby affecting glucose metabolism and cancer microenvironment [74]. Specifically, CRNDE promotes metabolic changes to shift energy production in cancer cells to aerobic glycolysis (the Warburg effect) [75]. Moreover, CRNDE can even be used as a scaffold for recruiting DMBT1 and c-IAP1 to stimulate PI3K-AKT pathway activity [76]. These findings further demonstrate the diverse functions of CRNDE.

**Fig. 5:**
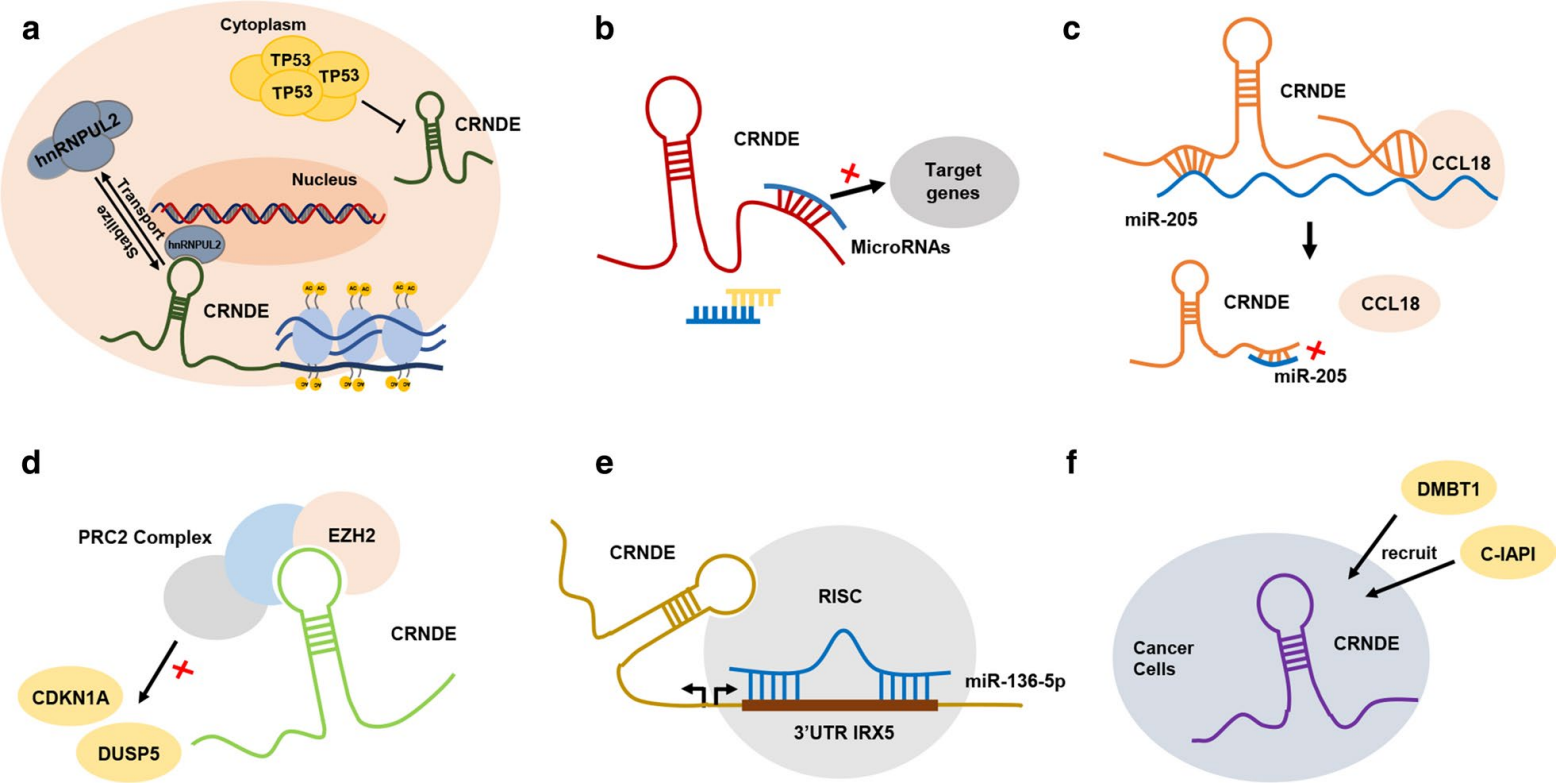
**a** The high specific expression of CRNDE in cancers may be a process of dynamic homeostasis. The histone acetylation in the promoter region accounts for the upregulation of CRNDE, hnRNPUL2 accumulating in the cytoplasm further stabilizes the mRNA level of CRNDE, while accumulation of the TP53 protein inhibits the expression of CRNDE. **b** CRNDE enhances tumorgenesis by acting as a molecular sponge or ceRNA via negatively targeting miRNAs, and then mediating its downstream target genes. **c** CRNDE binds to miR-205 and CCL18, and promotes cancers progression by sponging miR-205 and releasing CCL18. **d** CRNDE epigenetically suppresses the expressions of DUSP5and CDKN1A by binding to EZH2 (the key components of PRC2). **e** CRNDE impairs miR-136-5p expression in a RISC manner, and a reciprocal repression feedback loop is possible between CRNDE and miR-136-5p, while the neighboring mRNA of CRNDE is IRX5, which is a downstream target gene of miR-136-5p. **f** CRNDE acts as a scaffold to recruit the DMBT1 and c-IAP1 to help them make a difference

The elevated and specific expression of CRNDE in cancers may be a process of dynamic homeostasis. Histone acetylation in the promoter region accounts for the upregulation of CRNDE [19], hnRNPUL2 accumulation in the cytoplasm further stabilizes the mRNA level of CRNDE [35], while accumulation of the TP53 protein inhibits CRNDE expression [66] (Fig. 5). Based on the preceding exploring about CRNDE, we propose CRNDE as a multifunctional lncRNA and its different splice variants can provide specific functional scaffolds for regulatory complexes, such as PRC2 chromatin-modifying complexes, by which some mediators can navigate to target genes. In addition, the promoters of these genes are then regulated by epigenetic modification, accompanying with the associated biological phenotypes. In addition to its role in the recruitment of proteins involved in chromatin modifications, CRNDE has been recognized as a miRNA sponge, ceRNA, RNA signal, RNA scaffold or a component of the feedback loop with miRNAs and mRNAs (Fig. 5).

However, CRNDE has multiple transcript isoforms and thus the more functions need to be excavated. Moreover, because of the diversity of lncRNAs functions, CRNDE may exert effects opposite to those generated by its cancer-promoting role, making the study about CRNDE challenging and uncertain. Thus far, the secondary structure and the specific mechanism of CRNDE have not been fully elucidated. Most studies on CRNDE are limited to the regulation of its transcription levels and lack in-depth mechanistic research. CRNDE, as a widely recognized lncRNA with potential clinical value, requires increasingly comprehensive and in-depth study.

## Data Availability

The datasets used and/or analysed during the current study are available from the corresponding author on reasonable request.

## Abbreviations

CRNDE: Colorectal neoplasia differentially expressed
lncRNA: Long non-coding RNA
CRC: Colorectal cancer
HCC: Hepatocellular carcinoma
BC: Breast cancer
GC: Gastric cancer
RCC: Renal cell carcinoma
MAPK: Mitogen-activated protein kinase
hnRNPUL2: Heterogeneous nuclear ribonucleoprotein U-like 2
ceRNA: Competitive endogenous RNA
NSCLC: Non-small-cell lung cancer
PIWIL4: Piwi-like RNA-mediated gene silencing 4
TSCC: Tongue squamous cell carcinoma
PTC: Papillary thyroid cancer
Bax: Bcl2-associated X
EGF: Epidermal growth factor
EGFR: Epidermal growth factor receptor
TKI: Tyrosine kinase inhibitor
MM: Multiple myeloma
PRC2: Polycomb repressive complex 2
DUSP5: Dual-specificity phosphatase 5
E2F1: E2F transcription factor 1
EMT: Epithelial-mesenchymal transition
LPS: Lipopolysaccharide
TLR3: Toll-like receptor-3
5-Fu: 5-fluorouracil
OXA: Oxaliplatin
LAD: Lung adenocarcinoma
IGF: Insulin-like growth factor
